# Living with Xeroderma Pigmentosum: a qualitative study of the psychosocial challenges experienced by families of children with a rare skin disorder

**DOI:** 10.1186/s13023-026-04410-6

**Published:** 2026-06-05

**Authors:** Jordana McLoone, Kyra Webb, Kathy Tucker, Antoinette Anazodo, Denise Wilson, Michael Rtshiladze, Linda K. Martin

**Affiliations:** 1 School of Clinical Medicine, UNSW Medicine & Health, Discipline of Paediatrics, University of New South Wales, Sydney, NSW Australia; 2 Kids Cancer Centre, Sydney Children’s Hospital, Sydney, NSW Australia; 3 Hereditary Cancer Centre, Sydney Children’s Hospital, Sydney, NSW Australia; 4 Department of Dermatology, Sydney Children’s Hospital, Sydney, NSW Australia; 5 Melanoma Institute Australia, Sydney, NSW Australia; 6 Department of Plastic Surgery, Sydney Children’s Hospital, Sydney, NSW Australia; 7 The Daffodil Centre, The University of Sydney, A Joint Venture with Cancer Council NSW, Sydney, Australia

**Keywords:** Xeroderma pigmentosum, Rare diseases, Paediatric dermatology, Psychosocial impact, Family experience, Qualitative research, Skin cancer risk, Health information needs

## Abstract

**Background:**

Xeroderma Pigmentosum (XP), is a rare genetic condition characterised by extreme sensitivity to ultra violet (UV) radiation, conferring a 2,000- to 10,000-fold increased risk of developing melanoma and non-melanoma skin cancers. Families affected by XP face intense emotional strain, ongoing medical surveillance and intervention, and stringent lifelong adaptations to minimise UV exposure. Despite these challenges, little is known about the psychosocial burden experienced by children with XP and their immediate family members. This study explored the lived experiences and support needs, both medical and psychosocial, of families caring for a child with XP.

**Method:**

We conducted qualitative semi-structured interviews, in person or via Zoom, with parents and children affected by XP, examining diagnostic experiences, psychosocial impacts, care preferences, and informational needs. The interview guide was developed by a multidisciplinary expert panel. Eligible participants included parents of children with XP, and patients or siblings aged 5–18 years without intellectual disability. Participants were recruited via the Australian Paediatric XP Support Group, representing the full known XP cohort in Australia. Of the seven identified families, five contributed at least one parent participant. Among eight identified children, four were ineligible, and one was not enrolled. Interviews were audio-recorded, transcribed verbatim, and analysed using inductive, line-by-line coding in NVivo Pro. Descriptive statistics summarised participant demographics.

**Results:**

Eight parents (63% female, mean age 45 years) and three children (67% female, mean age 10 years) participated. Four themes emerged: (1) a prolonged and distressing diagnostic journey, often marked by misdiagnosis and uncertainty; (2) strong preferences for integrated, multidisciplinary care to reduce fragmentation; (3) significant psychosocial impacts, including isolation, anxiety-driven vigilance, and challenges adapting to absolute UV-protective routines; and (4) substantial unmet information needs at diagnosis, leaving families feeling overwhelmed and underprepared.

**Conclusion:**

Families affected by XP experience significant and enduring psychosocial burden. Findings highlight the urgent need for coordinated, interdisciplinary support that extends beyond medical care. This study contributes to the growing call within the rare disease community for integrated care models that centre patient and family wellbeing.

Xeroderma Pigmentosum (XP) is a rare autosomal recessive disorder characterised by defective DNA repair mechanisms, leading to extreme sensitivity to ultraviolet (UV) radiation [1, 2]. Affected individuals face a 10,000-fold increased risk of non-melanoma skin cancers and a 2,000-fold increased risk of melanoma compared to the general population, with the median first skin cancer diagnosis being under 10 years of age [3]. Beyond skin cancer, XP is a progressive disorder; up to 25% of affected individuals experience neurodegenerative complications including hearing loss, vision impairment, and motor deficits, which significantly diminish quality of life and life expectancy [2–4]. Children with XP often undergo repeated, invasive surgical procedures from a young age to remove both confirmed skin cancers and suspicious lesions [5]. These resections, critical for minimising disease progression, can result in substantial physical and emotional trauma, including facial disfigurement [5]. With a median life expectancy of 50 years for those with neurological involvement, XP represents a devastating diagnosis for both the affected child and their family [3, 6, 7].

The impact of XP is particularly pronounced in Australia, where high UV levels amplify both the medical and psychosocial burden of the condition [8–10]. While strict adherence to sun protection practices and regular clinical surveillance have been shown to significantly improve life expectancy, managing XP requires radical and sustained lifestyle modifications [5, 7]. Despite these demands, the broader psychosocial impacts remain underexplored, particularly the psychological, emotional, and financial toll on families, as well as the barriers to accessing appropriate information, services, and support within the context of a rare disease [11, 12].

Despite the high stakes and complex needs of families affected by XP, no research to date has specifically explored the psychosocial experiences of paediatric families. While previous work has documented the lifestyle adaptations required for photoprotection and efforts to maintain a sense of normality within affected families [13, 14], the psychological, emotional, and support-related dimensions remain largely unexamined. This study addresses this gap by exploring the lived experiences, care preferences, and psychosocial needs of families caring for a child with XP. Insights from this work aim to inform the development of holistic clinical services that integrate medical and psychosocial care, supporting better outcomes for this highly vulnerable population.

## Method

### Aim, design, and setting

This qualitative study aimed to explore the lived experiences, psychosocial impacts, and care preferences of families affected by XP, a rare genetic condition associated with extreme UV sensitivity and childhood-onset skin cancer risk. Semi-structured interviews were conducted between December 2023 and June 2024, either in person or via Zoom, depending on participant preference. The study was based in Australia and recruited nationally via the Australian XP Support Group, which encompasses all known Australian XP families.

### Participants

Eligible participants included: (1) parents or guardians of children diagnosed with XP under the age of 18, and (2) children aged 5–18 years with XP and no intellectual disability. Seven known XP families in Australia, with eight affected children, were approached via email. Interested families contacted the research team and were provided with the Participant Information Statement.

### Data collection

A semi-structured interview guide was developed based on a review of existing literature and consultation with a multidisciplinary expert panel comprising a paediatric dermatologist, dermatology nurse educator, geneticist, oncologist, plastic surgeon, and psycho-oncologist. The guide explored six domains: (i) diagnostic journey, (ii) current care, (iii) psychosocial impacts, (iv) prognostic concerns, (v) information and resource needs, and (vi) care preferences. Questions were adapted to be age-appropriate for children. All interviews were conducted by the first author (JM), a postdoctoral researcher with over 15 years of experience in qualitative and psycho-oncology research. Prior to the study interview, informed consent was obtained via a secure REDCap form and participants completed an online demographic questionnaire.

### Ethical considerations

The study received approval from the Sydney Children’s Hospital Network Human Research Ethics Committee (2023/ETH/02061). To monitor participant distress, the Distress Thermometer tool was administered before and after each interview. A rating > 7/10 or a change of > 3 points between pre- and post-interview scores prompted a follow-up discussion about psychological support options.

### Data analysis

Quantitative demographic data were analysed descriptively using IBM SPSS Statistics (v27; IBM Corp). Interviews were transcribed verbatim and analysed inductively using NVivo Pro (QSR International) [15], following the Miles and Huberman framework for thematic analysis [16]. Line-by-line coding was conducted to identify key concepts, which were grouped into nodes and refined into preliminary themes. Matrix coding was used to compare perspectives across parent and patient groups. Themes were iteratively reviewed and refined through collaborative team discussions, with triangulation among researchers used to enhance rigour and credibility.

## Results

### Demographic data

At the time of the study, the known Australian paediatric XP cohort consisted of seven families, of whom five participated. Two families did not express an interest in study participation. A total of eight parents (63% mothers) participated in an interview, with a mean age of 45 years (range: 32–62 years). This represented parents who cared for children six of the eight known XP children in Australia and represented subtypes XPC (*n =* 2) and XPD (*n* = 4). Prior skin cancer diagnosis and excisions were reported for one child. Three children with XP also participated in interviews (67% female), with a mean age of 10 years (range: 8–13 years). Reported subtypes among the children included XPC (*n =* 1) and XPD (*n* = 2). Of the remaining five children in the known Australia cohort who did not participate in an interview, two were from families who declined participation, two had an intellectual disability precluding participation, and one was below the eligible age threshold.

### Thematic analysis

Three inter-related themes were identified: (i) long journey to diagnosis; (ii) care preferences; (iii) psychosocial impact.

#### Theme 1: A long road to diagnosis, a longer path to adaptation

For most families, the path to an XP diagnosis was lengthy and convoluted, often characterised by misdiagnoses and delayed investigations. Common early misdiagnoses included eczema or general photosensitivity, with mothers frequently blamed for not adequately protecting their young children from the harsh Australian sun. Assumptions led to significant delays in identifying the underlying genetic condition and compounded parental distress. In the absence of a possible diagnosis, extensive medical tests were conducted with no further answers, sometimes years passing before XP was considered. Parents described this period as one of profound uncertainty and helplessness, reporting frustration with the lack of progress despite repeated efforts to seek medical advice:

*We kept asking every time I went to the dermatologist*,* is there any new sunscreen that we could try*,* because it’s unfathomable to think that sunscreen*,* plus zinc*,* plus long sleeves*,* plus a hat*,* plus sunnies*,* was not enough. You know*,* she would still get burnt - it might just be little burns – might just be little patches of redness and so for me was the constant anxiety of it ‘What’s she gonna come back to me like?’* [ID01, Parent]

A turning point often came when a second clinical opinion was sought, or when a severe sunburn or noticeable skin change prompted further investigation. Parents reflected on the emotional toll of this process, with many experiencing guilt and regret for not pursuing a diagnosis more aggressively, knowing that the sun damage accumulated during this time was irreversible:

*We both felt very guilty that we’d left it seven and a half years before we knew what was going on. [Child]’s got seven and a half years of skin damage already. Yeah*,* so that was hard to take.* [ID02, Parent]

Once XP was considered as a likely diagnosis (i.e., clinical diagnosis), parents often reported feeling shock and dread:

*[By then] I’d done a bit of research into it*,* and had thought no way like this*,* there’s no chance like…there’s like*,* the prognosis is terrifying… so when they said*,* like the words xeroderma pigmentosum*,* I was like ‘Oh my God.’* [ID05, Parent]

Alongside the medical diagnosis, families were advised to implement urgent lifestyle changes, often prior to genetic confirmation, which typically required an additional 6–12 months. Clinicians recommended avoiding all sun exposure, however, no specific thresholds (e.g., a safe UV value) were provided. Parents described this ambiguity as a significant source of burden, noting the stress of having to independently research and develop protective protocols in the absence of clear, evidence-based guidelines. Many expressed ongoing anxiety about whether their chosen strategies were sufficiently stringent to prevent skin damage or future malignancies. This uncertainty contributed to a pervasive fear that even minor lapses in protection might result in irreversible harm:

*…because you never know*,* especially when they’re young*,* you never know whether what you’re doing is enough.* [ID08, Parent]

This sense of urgency was contrasted with the extended adjustment period families reported was necessary to reestablish a sense of normality, control, and appropriate management of safe UV levels.

*[It has] taken us two years sort of to really establish where we can and can’t go or what we can and can’t do. And [getting the UV meter] was really eye opening. Because as soon as we had that*,* then we set out our safe number of what we keep [patient] under. And that really dictated where our lives went from there.* [ID05, Parent]

While clinicians facilitated genetic testing and provided disease-specific information, no families reported receiving adequate advice on modifying their lifestyle and environment to protect their child. Consequently, parents relied heavily on international XP communities with lived experience for practical guidance:

*We weren’t aware of anyone else in Australia that had an XP diagnosis. And so I’d sought out online and through social media had found a Mum in America. And she talked me through like what to get and what resources I needed to get* [ID05, Parent]

This self-guided approach created significant stress, as families of this rare disease felt they were “*on their own*,” forced to navigate complex decisions without sufficient support or contextually relevant information. For example, navigating the warmer months in Australia was particularly challenging:

*…in spring and summer it’s not possible*,* it’s just too much [to be dressed in protective clothing*,* hats*,* gloves] you can see you get suffocated*,* feel suffocated* [ID06, Parent]

Most families turned to international resources and support networks, particularly in the USA and Europe, for UV-protective clothing (e.g. from Germany), peer support, and guidance on educating extended family, schools, and communities. While invaluable, these resources were expensive due to currency conversion and shipping costs, operated across time zones, and were not always applicable to the local setting. Although the global XP community offered a sense of connection, the diversity of XP sub-types limited the relevance of shared advice. Parents were left to independently advocate for their child, navigate school modifications, and establish new daily protocols that reshaped family life, all while experiencing the emotional impact of the diagnosis. This period was characterised by urgency, isolation, an overwhelm of tasks, and uncertainty, with little formal support and constant internal questioning about whether their protective efforts would be enough.

In all cases, genetic testing confirmed the clinical diagnosis. Reported time to genetic confirmation was 3–9 months. Families reported satisfaction with their understanding of the condition, including its genetic basis, clinical symptoms, and potential long-term implications. While parents reported sufficient understanding of the variability across XP sub-types, this limited parents’ ability to find comparable cases, even within specialised support communities, further compounding feelings of uncertainty regarding how their child’s disease may progress.

#### Theme 2: Lack of integrated, family-centred models of care

Families expressed a strong preference for integrated models of care that centralised expertise across multiple disciplines. Parents emphasised the need for structured collaboration between dermatology, ophthalmology, neurology, and allied health professionals to comprehensively address the multisystemic and psychosocial complexities of XP. While dermatology services provided essential guidance on UV safety and skin cancer surveillance, parents reported that these services were not expert in their child’s broader needs, including neurocognitive concerns, developmental delays, and emotional wellbeing. Parents expressed concern that non-dermatology specialists in their child’s treating team lacked knowledge of XP and XP sub-type specific complications. They believed that care quality could be improved if these providers worked within a multidisciplinary team with dedicated XP expertise:

*…clinicians would understand why you’re there*,* and why you’re coming to an XP clinic. They know what you need to be looking out for.* [ID01, Parent]

Continuity of care emerged as a critical component of effective XP management. Parents emphasised the importance of consistently seeing the same healthcare provider, believing that familiarity with their child’s baseline skin presentation was essential for detecting subtle clinical changes over time:

*…you need the consistency of your clinicians*,* who know you*,* when you’ve got something changing over time*,* both from a dermatology point of view and from a neurological point of view.* [ID02, Parent]

Allied health was frequently cited as a significant unmet need. Occupational therapy and physiotherapy were seen as valuable for addressing developmental and physical challenges, but families struggled to attend additional appointments amid demanding medical surveillance schedules, daily care routines, and financial constraints. The need to attend services in UV-safe settings, preferably at home, added further complexity. While families wanted to *“do it all”*, even services perceived as beneficial were often deprioritised due to the cumulative burden:

*We took (our child) for counselling as well*,* psychological counselling. But it was just too much for us.* [ID07, Parent]

Families consistently described the psychological toll of XP as extending beyond the affected child, with parents and siblings often feeling unsupported. The emotional impact of XP on the wider family unit was substantial, underscoring the need for comprehensive, family-centred psychosocial support services:

*You have to live like that… whether it’s sunny weather*,* it’s cloudy rain. When it is every day without a break*,* then it has become very hard… and that’s a lot to take away from anybody. And so this condition*,* it’s not only him that that suffers*,* it’s everybody in the family.* [ID06, Parent]

Geographic inequalities further exacerbated these challenges for families living in rural areas. Long travel distances, lack of local XP expertise, and the burden of educating healthcare providers unfamiliar with the condition were recurrent themes. Parents described the time and effort involved in advocating for their child and ensuring appropriate care:

*We spend so much time explaining XP to doctors who have never seen it before. It takes up the whole appointment*,* and we don’t get the help we came for.* [ID04, Parent]

Finally, families also reported considerable difficulties accessing disability-related financial support, particularly through national schemes where assessors lacked awareness of XP. Multiple rejections and prolonged administrative processes compounded the already significant emotional and logistical load:

*…it took months and months to get extra documentation…oh gosh it was such a process* [ID02, Parent]

#### Theme 3: Psychosocial impact

The emotional, social, and psychological toll of managing XP was profound, affecting every member of the family. Parents described chronic worry about their child’s safety, grief over the loss of a “normal” childhood, and the relentless mental load of managing the condition. For children, the constant lifestyle restrictions, fear of developing skin cancer, and social isolation deeply affected their self-esteem and overall well-being. One parent reflected:

*[My child] will be sitting inside by himself*,* and he will*,* he’s emotional*,* he will come to me all the time*,* ‘oh the kids are playing outside*,* I’m just sitting here*,* what should I do? *[ID06, Parent]

Participants also reported concerns about the ongoing impact of essential medical procedures, particularly those involving skin cancer removal. One parent described how these procedures had affected their child:

*It’s been a long journey but*,* every couple of months he’s had surgeries*,* a lot of skin cancer removal. That gets hard. He gets traumatised every time he comes in now.* [ID03, Parent]

Siblings were also deeply affected, some experiencing resentment due to the disproportionate attention given to their sibling with XP, while others became overly protective. Parents noted the strain the condition placed on their own relationships, with one describing it as *“a constant pressure cooker”* of stress and responsibility.

Social isolation was a recurring theme, with both children and families describing limited opportunities to engage in normal activities. Parents often struggled to find community groups willing to accommodate their child’s needs. For children, this lack of participation in sports, school outings, or social events contributed to feelings of exclusion:

*He stopped asking to go to birthday parties because he knew we’d say no. It’s easier for him not to ask than to hear ‘no’ again.* [ID06, Parent]

A diagnosis of XP was also associated with persistent uncertainty about the future. Parents described living with a pervasive fear that even the most meticulous planning and protective measures might ultimately be insufficient. This sense of fragility extended across years, as families grappled with the distressing possibility that their current actions could irreversibly shape their child’s cancer risk decades into the future. One parent captured this enduring and pervasive anxiety:

*We just had to make some environments for the (child) safe. There’s literally not*,* not even the house wasn’t safe…There was nowhere in the world that was safe.* [ID02, Parent]

Parents reported constant vigilance of their child’s environment to ensure safety, identifying the mental load of determining whether an outing was possible, or a location was safe. While this vigilance afforded some sense of control and confidence in preventing harm, parents of children with subtypes prone to progressive neurological involvement expressed feeling a lack of control and ongoing uncertainty. These parents often described greater unmet information and support needs:

*It’s not even so much the skin cancer; it’s everything else. With XPD*,* it can be neurological—hearing loss*,* vision loss*,* your balance*,* like gross motor*,* fine motor. That’s what terrifies me. I think we’re doing pretty well protecting [child]’s skin. But the other stuff*,* we have absolutely no control over.* [ID05, Parent]

As time progressed following their child’s diagnosis, parents reported gaining confidence in managing XP and maintaining a UV-safe environment for their child. However, parents reported anxiety about the future, particularly the prospect of their child assuming responsibility for risk management in their teenage years. Parents recognised that control over sun-safety measures would shift and their teen may not wish to be different from their peers or miss out on social events, choosing a “normal life” over long term health benefits. One parent reflected:

*It’s a bit scary when she’s going to be out on her own*,* living her own life*,* making her own decisions. Like I hope that we have trained her and given her enough knowledge and set her on the pathway.* [ID02, Parent]

Despite these challenges, some families developed effective coping strategies, such as creating structured routines, building peer support networks, and finding small ways to normalise daily life. One family reflected on the first year following their child’s diagnosis and the impact of missed activities on their child. After assessing and questioning *“how do we make this safe?”*, the family committed to designing a bespoke hood to enable participation in outdoor sports and modified wetsuits to compete in the school swimming carnival. Another source of resilience for parents was connection with others who understood the condition:

*When we met up*,* it was amazing. But yeah*,* I suppose the XP can feel a bit like you’re drowning sometimes. So it’s just sometimes it’s one foot in front of the other.* [ID05, Parent caring for a child with XP]

## Discussion

This study provides critical insights into the lived experiences, care preferences, and psychosocial impacts of families managing Xeroderma Pigmentosum (XP) in Australia. By examining the challenges families face across diagnostic, medical, and psychosocial domains, these findings address a notable gap in the rare disease literature. The results reinforce the urgent need for tailored, multidisciplinary models of care that reflect the multisystemic complexity of XP and place families at the centre of care delivery.

From the outset, families described a protracted and distressing diagnostic journey. Misdiagnoses and delays were common, with many families engaging with multiple health professionals before XP was considered. Once a diagnosis was established, it was often met with distress and regret, particularly due to concerns that irreversible UV damage had already occurred in this time. These experiences reflect well-documented systemic issues in rare disease care, where diagnostic delays are common and carry significant emotional and medical consequences [17]. In an Australian study of families affected by rare diseases, diagnostic delays were associated with high levels of stress, anxiety, inappropriate treatment, and uncertainty about reproductive planning [17]. In the context of XP, such delays are particularly concerning given the direct link between early sun exposure and future skin cancer risk [6] highlighting the critical need to promote earlier recognition and symptom awareness among healthcare providers.

Families expressed a clear preference for integrated, multidisciplinary models of care that could bring together dermatology, neurology, ophthalmology, allied health, and psychosocial support in a coordinated, UV-safe setting. A single-day, co-located clinic model was seen as an ideal solution to the logistical burden of managing multiple appointments, especially for those living rurally. These findings are consistent with broader research in rare diseases, which shows that families strongly favour care models that minimise fragmentation and reduce the stress of navigating complex health systems [18]. Despite the need for allied health support—including psychological care and occupational/physiotherapy—these services were frequently deprioritised due to competing demands, limited local availability, concerns about UV exposure when attending appointments, and costs. Similar challenges have been reported in families of children with complex chronic conditions, where the uptake of psychosocial screening is high, but access to care remains limited unless services are embedded within medical appointments [19].

Beyond structural aspects of care, this study underscores the enduring psychosocial toll of XP on both patients and family members. Participants described profound uncertainty, grief, and self-doubt during the post-diagnostic period, compounded by a lack of access to practical and emotional support. Although most parents reported adequate understanding of the genetic basis of XP, they identified an absence of guidance on implementing UV-safe protocols, managing their fear of cancer occurrence, and adjusting to the lifelong implications of the condition. This is consistent with findings from other rare disease populations, where a lack of disease-specific information and uncertainty in its management contributes to poor psychological outcomes and caregiver strain [20–23]. Tailored psychological interventions are urgently needed, particularly during vulnerable periods such as diagnosis and transition to self-management during adolescence and early adulthood.

Together, these findings provide a compelling rationale for the development of XP-specific care pathways that integrate medical, psychosocial, and practical support. As with other rare diseases, effective XP care must extend beyond clinical surveillance to address the daily realities families face—balancing risk, uncertainty, and the desire to preserve quality of life.

### Strengths and limitations

This study is the first, to our knowledge, to comprehensively explore the psychosocial and care-related experiences of families managing XP in a national cohort. Inclusion of both parent and patient perspectives enriched the data and provided a nuanced understanding of XP’s impact across the family unit. However, several limitations should be acknowledged. The sample size, although reflective of the condition’s rarity, limits the generalisability of findings. Also, the structure of the Australian healthcare system and the country’s high UV environment may limit the transferability of findings to other geographic or healthcare settings.

### Clinical implications

These findings have several important implications for clinical practice and health system design. First, the establishment of centralised, multidisciplinary clinics for children with XP could substantially reduce the burden of fragmented care, while addressing the unique medical and psychosocial needs of affected families. Multidisciplinary clinics have been shown to be cost effective, for example, in the United Kingdom [24]. Such clinics should integrate dermatology, ophthalmology, neurology, allied health, and dedicated psychological support for both patients and their families.

Second, targeted education for healthcare providers is essential to improve early recognition of XP, potentially reducing diagnostic delays and limiting avoidable UV-related harm. Third, the development of locally relevant, evidence-based resources, such as UV protection protocols and XP-specific educational materials, would reduce reliance on parents forming international networks which risk the spread of misinformation and would improve care consistency.

Finally, structural reform is needed to improve access to financial and social support. Greater awareness of XP among disability scheme assessors, and broader eligibility criteria for psychosocial support programs currently limited to individuals with a confirmed cancer diagnosis, is essential. Families affected by XP experience considerable psychological burden due to their child’s 10,000-fold increased cancer risk relative to their peers, and should not be excluded from systems designed to mitigate serios health-related disadvantage.

Rare conditions like XP expose critical cracks in our current models of care—where rigid systems, low awareness, and a lack of coordinated services leave families navigating extreme complexity with little support. Addressing these gaps requires not only clinical expertise, but also system-level compassion, agility, and investment in multidisciplinary rare disease frameworks. Despite our best efforts, recruitment for this study was limited—a common challenge in rare disease research. Stronger national and international collaboration, including shared protocols with other light-sensitive conditions, may support future research efforts to reach saturation and better define care pathways.

## Conclusion

This study underscores the complex and enduring challenges faced by families caring for a child with Xeroderma Pigmentosum (XP), revealing critical gaps in diagnostic pathways, care coordination, and psychosocial support. By centring the perspectives of families, our findings offer important direction for the design of more integrated, responsive, and equitable care models. Addressing these unmet needs will not only improve health and psychosocial outcomes but also contribute to broader efforts to strengthen rare disease care systems through patient- and family-informed approaches.

## Data Availability

The datasets generated and/or analysed during the current study are highly sensitive and are not publicly available as per the conditions of our ethics and governance approvals.

## References

[1] van Steeg H, Kraemer KH. Xeroderma pigmentosum and the role of UV-induced DNA damage in skin cancer. Mol Med Today. 1999;5(2):86–94. 10200950 10.1016/s1357-4310(98)01394-x

[2] Kraemer KH, Lee MM, Scotto J. Xeroderma pigmentosum: cutaneous, ocular, and neurologic abnormalities in 830 published cases. Arch Dermatol. 1987;123(2):241–50. 3545087 10.1001/archderm.123.2.241

[3] Bradford PT, Goldstein AM, Tamura D, Khan SG, Ueda T, Boyle J, et al. Cancer and neurologic degeneration in xeroderma pigmentosum: long term follow-up characterises the role of DNA repair. J Med Genet. 2011;48(3):168–76. 21097776 10.1136/jmg.2010.083022 PMC3235003

[4] Garcia-Moreno H, Langbehn DR, Abiona A, Garrood I, Fleszar Z, Manes MA, et al. Neurological disease in xeroderma pigmentosum: prospective cohort study of its features and progression. Brain. 2023;146(12):5044–59. 38040034 10.1093/brain/awad266 PMC10690019

[5] Moriwaki S, Kanda F, Hayashi M, Yamashita D, Sakai Y, Nishigori C, et al. Xeroderma pigmentosum clinical practice guidelines. J Dermatol. 2017;44(10):1087–96. 28771907 10.1111/1346-8138.13907

[6] Lucero R, Horowitz D, Xeroderma Pigmentosum. StatPearls. StatPearls Publishing LLC. Treasure Island, FL, USA; 2020. 31855390

[7] Paolino A, Keogh R, Langan SM, Lehmann AR, Garrood I, Fassihi H, editors. Life Expectancy of Xeroderma Pigmentosum Patients: Evidence from a UK Study. WILEY 111 RIVER ST, HOBOKEN 07030 – 5774, NJ USA: JOURNAL DER DEUTSCHEN DERMATOLOGISCHEN GESELLSCHAFT; 2025.

[8] Sneyd MJ, Cox B. A comparison of trends in melanoma mortality in New Zealand and Australia: the two countries with the highest melanoma incidence and mortality in the world. BMC Cancer. 2013;13(1):372. 23915380 10.1186/1471-2407-13-372 PMC3750694

[9] Arnold M, Singh D, Laversanne M, Vignat J, Vaccarella S, Meheus F, et al. Global Burden of Cutaneous Melanoma in 2020 and Projections to 2040. JAMA Dermatol. 2022;158(5):495–503. 35353115 10.1001/jamadermatol.2022.0160 PMC8968696

[10] Laouali S, Adam ND, Issaka H, Maïmouna OM, Sani LIM, Doulla M, et al. Xeroderma pigmentosum: twelve cases at the National Hospital of Niamey, Niger. Our Dermatology Online/Nasza Dermatologia Online. 2023;14(1).

[11] Sarkany R, Norton S, Canfield M, Morgan M, Foster L, Sainsbury K, et al. Identifying the psychosocial predictors of ultraviolet exposure to the face in patients with xeroderma pigmentosum: a study of the behavioural factors affecting clinical outcomes in this genetic disease. J Med Genet. 2022;59(11):1095–103. 35393336 10.1136/jmedgenet-2021-108323 PMC9613853

[12] Sethi M, Lehmann AR, Fassihi H. Xeroderma pigmentosum: a multidisciplinary approach. EMJ Dermatol. 2013;1:54–63.

[13] Anderson R, Walburn J, Morgan M. Experiences of stigma over the lifetime of people with xeroderma pigmentosum: A qualitative interview study in the United Kingdom. J Health Psychol. 2019;24(14):2031–41. 28810476 10.1177/1359105317714643

[14] Morgan M, Anderson R, Walburn J, Weinman J, Sarkany R. The influence of perceived medical risks and psychosocial concerns on photoprotection behaviours among adults with xeroderma pigmentosum: a qualitative interview study in the UK. BMJ open. 2019;9(2):e024445. 30782905 10.1136/bmjopen-2018-024445 PMC6377541

[15] Lumivero. NVivo (Version 12). 2017. https://www.lumivero.com.

[16] Miles MB. Qualitative data analysis: an expanded sourcebook. Thousand Oaks. 1994.

[17] Zurynski Y, Deverell M, Dalkeith T, Johnson S, Christodoulou J, Leonard H, et al. Australian children living with rare diseases: experiences of diagnosis and perceived consequences of diagnostic delays. Orphanet J Rare Dis. 2017;12(1):68. 28399928 10.1186/s13023-017-0622-4 PMC5387276

[18] Walton H, Simpson A, Ramsay AIG, Hunter A, Jones J, Ng PL, et al. Development of models of care coordination for rare conditions: a qualitative study. Orphanet J Rare Dis. 2022;17(1):49. 35164822 10.1186/s13023-022-02190-3 PMC8843018

[19] Marshall KH, Pincus HA, Tesson S, Lingam R, Woolfenden SR, Kasparian NA. Integrated psychological care in pediatric hospital settings for children with complex chronic illness and their families: a systematic review. Psychol Health. 2024;39(4):452–78. 35635028 10.1080/08870446.2022.2072843

[20] Kenny T, Bogart K, Freedman A, Garthwaite C, Henley SM, Bolz-Johnson M, et al. The importance of psychological support for parents and caregivers of children with a rare disease at diagnosis. Rare Disease Orphan Drugs J. 2022;1(2):N/A-N/A

[21] Smits RM, Vissers E, Te Pas R, Roebbers N, Feitz WF, van Rooij IA, et al. Common needs in uncommon conditions: a qualitative study to explore the need for care in pediatric patients with rare diseases. Orphanet J Rare Dis. 2022;17(1):153. 35379257 10.1186/s13023-022-02305-w PMC8981675

[22] Spencer-Tansley R, Meade N, Ali F, Simpson A, Hunter A. Mental health care for rare disease in the UK–recommendations from a quantitative survey and multi-stakeholder workshop. BMC Health Serv Res. 2022;22(1):648. 35568910 10.1186/s12913-022-08060-9 PMC9107210

[23] Nasrallah NA, Wiese BM, Sears CR. Xeroderma pigmentosum complementation group C (XPC): Emerging roles in non-dermatologic malignancies. Front Oncol. 2022;12:846965. 35530314 10.3389/fonc.2022.846965 PMC9069926

[24] Fassihi H, Garrood I, Chandler N, Mohammed S, Lehmann AR, Sarkany R. Xeroderma Pigmentosum in the UK. In: Nishigori C, Sugasawa K, editors. DNA Repair Disorders. Singapore: Springer Singapore; 2019. pp. 99–114.

